# Predicting the distribution of *Deyeuxia angustifolia* habitats in the Tumen River Basin due to climate and land-use changes

**DOI:** 10.3389/fpls.2026.1865223

**Published:** 2026-06-26

**Authors:** Jiayuan Zhang, Yuxi Peng, Zhen Wang, Wenxuan Yu, Chongzhe Zhong, Chengbin Yang, Yuqi Liu, Guanglan Cao

**Affiliations:** 1 College of Geography and Ocean Science, Yanbian University, Hunchun, Jilin, China; 2 School of Natural Resources, Faculty of Geographical Science, Beijing Normal University, Beijing, China; 3 College of Environmental Science and Engineering, Nankai University, Tianjin, China

**Keywords:** climate change, habitat, land-use change, species distribution model, wetland

## Abstract

Habitat dynamics of the representative wetland plant, *Deyeuxia angustifolia* serve as a key indicator of ecosystem health in the transboundary Tumen River Basin. To investigate spatiotemporal evolution under combined climate and land-use changes, we integrated multi-source environmental variables into a species distribution model (SDM) using the Google Earth Engine (GEE) cloud platform. Current habitat suitability was simulated, and distributions for the 2050s were projected under three shared socioeconomic pathways (SSP126, SSP245, and SSP585). Currently, high and moderate suitability habitats cover 25.5% of the basin, concentrated in Russia’s khasan District, China’s Wangqing County, and Ryanggang Province in the Democratic People’s Republic of Korea. Future scenarios suppress distribution; however, as emission concentrations rise, habitat loss areas decreased, with land-use change emerging as the primary driver of expansion. Habitat changes were most pronounced in China, under the low-emission SSP126 scenario. Conversely, under SSP585, suitable areas remain the largest among future projections, though still below current baselines. This study coupled climatic and anthropogenic variables and addressed previous modeling limitations. The study findings provide scientific support for mitigating wetland degradation, conserving biodiversity, and guiding ecological management in the Tumen River Basin.

## 1 Introduction

Wetlands are biodiversity hotspots that provide essential habitats for globally endangered and endemic species, playing a critical role in biodiversity conservation (Wu et al., 2019). However, these ecosystems have undergone substantial ecological transformations owing to climate change and human activities (Xiong et al., 2023). Climate change persistently affects the habitats of species through various pathways. First, it directly affects environmental factors within wetlands, such as water temperature and soil element content (Guan et al., 2025), thereby affecting processes such as the carbon cycle in wetland ecosystems (Wang et al., 2025), which subsequently challenges species survival (Wang et al., 2025). Additionally, climate change can shift species distributions by altering interspecific interactions and the physical structure of habitats (Li et al., 2024). These effects accelerate changes in species distribution and abundance, potentially threatening ecosystem functions and biodiversity. As these changes vary considerably across spatial and temporal scales, understanding the exact effects of climate change on species habitats is crucial for effective conservation (Lu et al., 2024).

Furthermore, amid ongoing global socioeconomic development, the rapid intensification of anthropogenic activities, including industrialization and urbanization, has profoundly influenced ecological stability. For instance, the loss and fragmentation of natural water habitats, changes in water quality, and agricultural expansion contribute to the degradation of wetland ecosystem functions, ultimately threatening biodiversity (Dawuda et al., 2024; Feng et al., 2016; Kayendeke et al., 2024; Galatowitsch, 2018).

*Deyeuxia angustifolia*, a native dominant wetland plant extensively distributed across the Sanjiang Plain in China (An et al., 2018), the Democratic People’s Republic of Korea (DPRK), and the Russian Far East, demonstrated remarkable environmental adaptability (Li et al., 2023). This species has a well-developed root system, high environmental tolerance, and strong reproductive capacity, enabling it to rapidly colonize diverse wetland habitats (Dong et al., 2023). Owing to these characteristics, *D. angustifolia* can substantially alter, or even disrupt, the structure and ecological functions of wetland ecosystems. For example, they may compete for resources or modify the soil microbial environment (Ni et al., 2021), thereby suppressing native shrub growth and reproduction. This can result in a reduction in species richness and biodiversity within native plant communities, ultimately affecting the overall function of wetland ecosystems (Li et al., 2017). Consequently, shifts in the habitat distribution of *D. angustifolia* could serve as key indicators of the health status of wetlands. Therefore, comprehensive studies and monitoring of habitat dynamics are crucial to provide scientific support for wetland conservation and management.

Currently, studies on *D. angustifolia* primarily focus on its physiological characteristics, functional traits, community composition, responses to high temperatures and nitrogen deposition, and effects on soil nutrient dynamics and enzyme activities (Jin et al., 2016; Zhang et al., 2022; Zong et al., 2016). However, research predicting its future habitat distribution remains limited (An et al., 2018; Jin et al., 2016; Zong et al., 2016). Most studies have focused on habitat changes under current climate models and future greenhouse gas emission scenarios, providing valuable theoretical insights for wetland conservation (Fedorov et al., 2024; Karichu et al., 2022; Wang Y. et al., 2022). Nevertheless, challenges such as limited data accuracy and modeling complexity persist. Given the intensifying impacts of human activities, research on *D. angustifolia* habitats should adopt a broader perspective that incorporates socio-economic factors beyond mere climate change. Therefore, enhancing the reliability and accuracy of habitat predictions by integrating both natural and anthropogenic variables should be the primary direction for future research.

With the continuous development of Earth observation big data, cloud computing, the Internet of Things, and remote sensing technologies, geospatial analysis and computing cloud platforms, such as the Google Earth Engine (GEE), now enable easy access to high-performance computing resources for processing large geospatial datasets, thereby addressing notable societal issues such as deforestation, drought, and water resource management (Kazemi Garajeh et al., 2024; Gorelick et al., 2017; Li et al., 2022; Zhao et al., 2024), making large-scale predictions of wetland habitat changes highly feasible. For example, Yi et al. (2024) used GEE combined with climate data and the Soil-Adjusted Vegetation Index (SAVI) to monitor the spatiotemporal dynamics of salt marsh vegetation, demonstrating superior efficiency and accuracy compared to traditional methods. Similarly, Wang M. et al. (2022) employed a Random Forest (RF) classifier within the GEE framework to analyze 35 years of wetland changes in Zhuhai City, significantly improving classification accuracy. Additionally, Mahdianpari et al. (2019) leveraged GEE to develop a dynamic wetland inventory map for Newfound-land. These studies provide valuable theoretical support for wetland resource conservation based on GEE, underscoring the platform’s immense potential and broad application prospects in wetland monitoring and management.

As species occurrence data continue to expand, occurrence-based species distribution models (SDMs) have emerged as an established research approach (Adde et al., 2023). SDMs are statistical or machine learning models that use known species occurrence data and as-sociated environmental variables to estimate a species’ ecological niche requirements and project them across defined spatial and temporal scales. These models are used to assess habitat suitability or predict the probability of species occurrence within their potential range, facilitating the designation and restoration of protected areas and the evaluation of threats posed by invasive species Angelieri et al., 2016; Frans et al., 2022). Currently, SDMs are widely applied and include Generalized Linear Models (GLM), Gradient Boosting Models (GBM), RF, and Maximum Entropy (MaxEnt). Specifically, MaxEnt is particularly notable for its high prediction accuracy and broad applicability, making it a frequently used tool for the conservation of endangered species and management of invasive species (Engler et al., 2012; Briscoe Runquist et al., 2019).

Furthermore, the application of SDMs on the GEE platform simplifies the modeling process and enhances computational efficiency. For example, Crego et al. (2022) demonstrated a streamlined workflow for constructing SDMs using occurrence data on GEE to analyze potential species distribution. However, applications combining GEE-based Random Forest models with both climatic and anthropogenic variables to predict future wetland species dynamics remain limited. Addressing this gap is critical for highly adaptable species like *D. angustifolia*, whose distribution is deeply inter-twined with both climate change and regional land-use alterations.

This study examines the transboundary Tumen River Basin as a critical yet vulnerable ecological zone, where *D. angustifolia* dynamics serve as key indicators wetland health. Using high-resolution Landsat imagery on the GEE platform, we integrated environmental variables, including current and future climate scenarios and land-use changes, to construct a GEE-based SDM with the RF algorithm. The objectives of this study are threefold: (1) to simulate the habitat conditions of *D. angustifolia* in 2020 and predict habitat changes and suitability under multiple scenarios for 2050; (2) to identify key factors driving distribution changes and assess how climate change and human activities jointly influence wetland ecosystems; (3) to provide scientific support for mitigating wetland degradation in the Tumen River Basin and promoting sustainable wetland conservation and management.

## 2 Materials and methods

### 2.1 Study area

**Figure 1:**
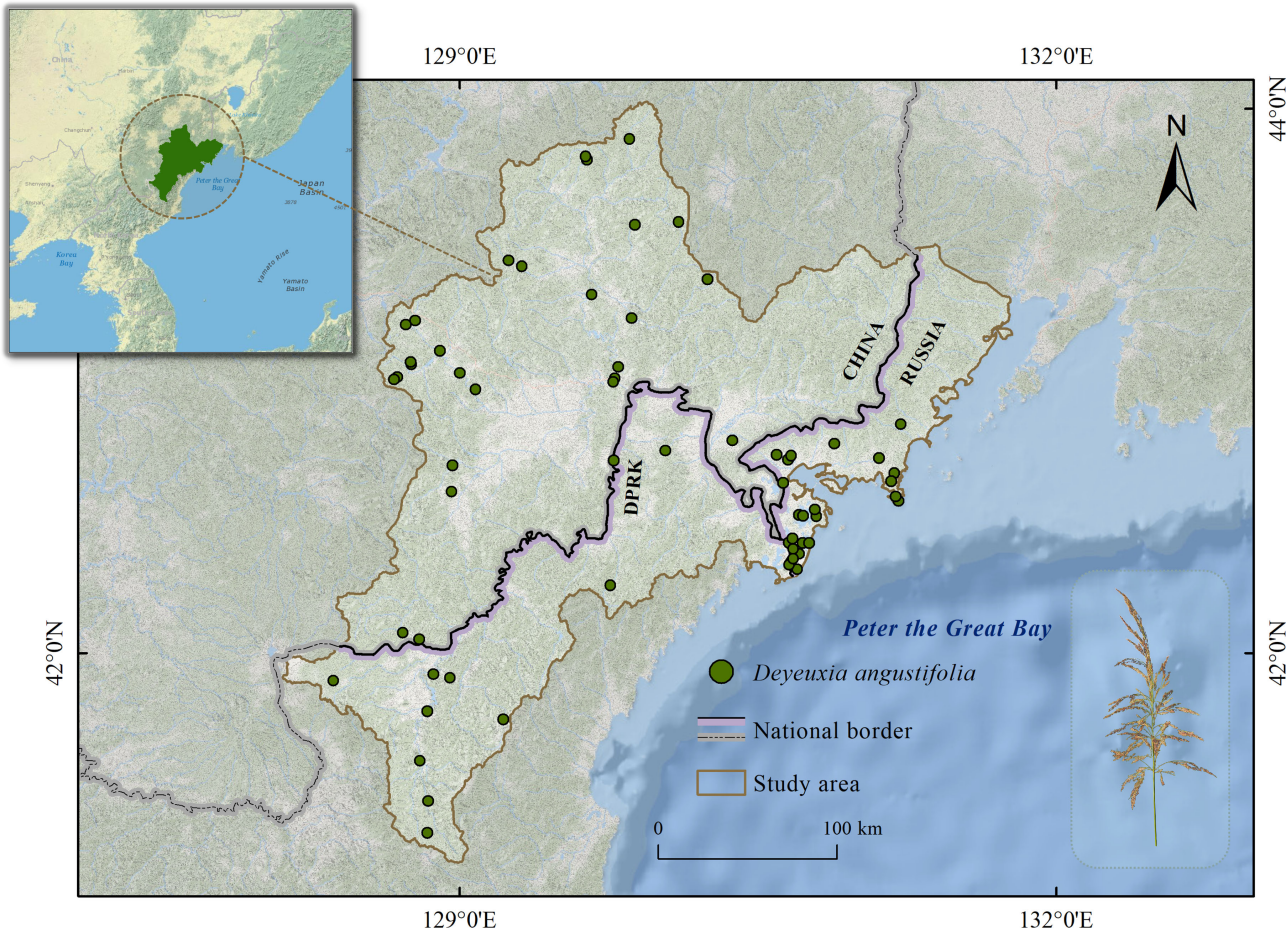
Study area and geographic distribution records of Deyeuxia angustifolia within the Tumen River Basin.

The Tumen River Basin (spanning from 128°06′ to 131°47′ E and from 41°13′ to 44°01′ N) covers approximately 37,400 km^2^ at the border tripoint of China, the DPRK, and Russia. Originating in the Changbai Mountains, the 521-km main stream flows primarily eastward, featuring an elevation gradient that descends from west to east. The basin experiences a temperate continental monsoon climate with distinct maritime features owing to its proximity to the Sea of Japan. The average annual temperature is ~5.3 °C, with an annual precipitation of 538 mm (Ding et al., 2024; Jin and Zhang, 2024). The basin is characterized by diverse wetland ecosystems and primary forests, such as Changbai larch and broadleaf mixed forests (Wang et al., 2023). As a representative natural complex in northern Eurasia, these habitats support rich biodiversity, including globally endangered species like Amur tigers and red-crowned cranes (Zong et al., 2022). However, climate change and anthropogenic activities have recently driven severe wetland soil degradation, significantly reducing natural areas and impairing regional ecological functions (Yang et al., 2019; Zheng et al., 2017) (**Figure 1**).

### 2.2 Materials

#### 2.2.1 Occurrence data

The *D. angustifolia* occurrence data were obtained from field surveys and public dataset. Field surveys were conducted from the Tumen River wetland patches between July and September 2017 using a grid sampling method, recording geographic coordinates by GPS. Additionally, publicly available data were primarily obtained from the Global Biodiversity Information Facility, which provides observation records for *D. angustifolia* (https://doi.org/10.15468/dl.6x462b). A total of 137 distribution points for *D. angustifolia* were initially collected, with data covering the years 1900–2021.

Approximately 70% of the records include data obtained after 1970 and they align well with the environmental baseline. The pre-1970 historical records are primarily located in transboundary regions, which have experienced very low anthropogenic land-use changes. The native ecological environments remain highly stable (Yu and Li, 2022; Li et al., 2024). Therefore, these early records accurately represent the inherent suitable habitats of the species. We retained these historical data to prevent niche truncation and baseline shifts (Faurby and Araújo, 2018; Monsarrat et al., 2019). Furthermore, a sensitivity analysis using only post-1970 data confirmed our choice. Retaining the full dataset prevents local spatial omission errors at the transboundary range edges (**Supplementary Figures 2**, **3**).

To avoid spatial clustering effects in the model predictions, we used the R package spThin to spatially thin the occurrence points for all species, setting the minimum neighbor distance threshold to 1 km. This process excluded data from urban construction areas, resulting in 62 adjusted distribution points for *D. angustifolia* (**Supplementary Table 3**).

#### 2.2.2 Environment data

Environmental variables were classified into four categories: topographic, climatic, natural, and human activities. Topographic data, including elevation, slope, and aspect, were obtained from the NASA SRTM Digital Elevation Model (30 m), provided by the GEE. The corresponding elevation, slope, and aspect bands were extracted using GEE. Climatic data comprising 19 bioclimatic variables were obtained from the WorldClim database (**Supplementary Table 1**). Current climate data were based on historical climate records from 1970 to 2000 and included precipitation and temperature values. Future climate data were selected from the Coupled Model Intercomparison Project Phase 6 (CMIP6), with 14 currently available simulation models assessed (**Supplementary Table 2**). These data were processed using the R package “terra” and exported for the SSP126, SSP245, and SSP585 emission concentration scenarios.

Natural factors included vegetation type and distance to rivers. The vegetation data were obtained from the GLC_FCS30 Global Land-Cover (30 m) dataset and reclassified into nine vegetation types: (1) evergreen broadleaf forest, (2) deciduous broadleaf forest, (3) evergreen coniferous forest, (4) deciduous coniferous forest, (5) mixed forest, (6) shrubland, (7) grassland, (8) moss-lichen, and (9) bare land. The distance to rivers was obtained from the OpenStreetMap (OSM) hydrological data, and distances to rivers and Tianchi Lake were calculated using the “Euclidean Distance” tool in ArcMap 10.8.

**Figure 2:**
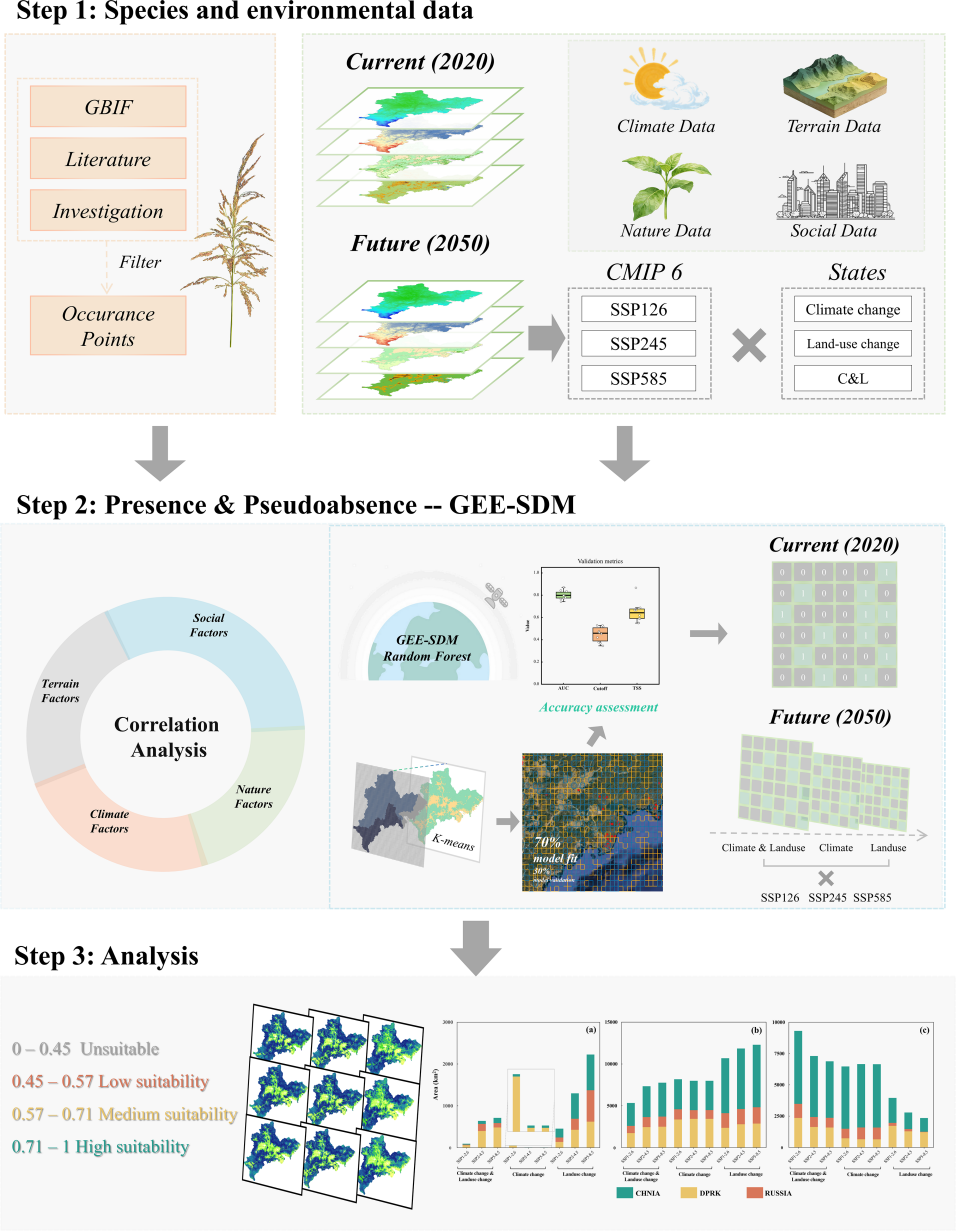
Study framework.

Human activity factors included the distance to built-up areas. Current scenario data were based on the GLC_FCS30 dataset, whereas future scenario data were obtained from the SSP-RCP scenario-based global land use projection dataset (2020–2100) at a resolution of 1 km (Zhang et al., 2023). This specific dataset was selected due to its robust simulation accuracy (Kappa = 0.94, Overall Accuracy = 0.97) and its high regional applicability. Unlike traditional global datasets, this framework utilizes the GCAM model to divide the globe into 235 water-basin level regions, which effectively captures the spatial heterogeneity and socio-economic diversity suitable for basin-scale studies like the Tumen River basin. Furthermore, the future urban area demands within this dataset were explicitly calibrated using projected GDP and population dynamics, ensuring that the socio-economic conditions were inherently adapted. The built-up land category was extracted, and the “Euclidean Distance” tool was used to calculate distances. All environmental variables used in this study were standardized to a spatial resolution of 1 km in the GEE, with the geo-graphic coordinate system standardized to WGS1984 (**Figure 2**, STEP 1).

### 2.3 Methods

#### 2.3.1 Model preparation

High correlations between environmental variables can lead to overfitting in species distribution models (SDMs). To ensure accurate model predictions and reduce col-linearity, we pre-ran the selected environmental data 10 times. Pearson’s correlation analysis was conducted on 19 bioclimatic variables, one natural variable, one land-use variable, and three topographic variables using the R package corrplot, and a correlation heatmap was generated. As categorical variables, soil factors were excluded from the correlation analysis. Environmental variables with a correlation coefficient >0.8 were excluded. The final variables selected for modeling were Aspect, Slope, Bio1, Bio12, Distance to the construction area, and Vegetation (**Supplementary Figure 1**).

For effective training of the SDM-random forest model, the background point environmental characteristics were categorized for the grid of the study area. These were primarily divided into habitat points similar to *D. angustifolia* presence points (presence) and habitat points resembling the absence points (absence). Owing to the challenges in obtaining the true absence data for *D. angustifolia*, pseudo-absence points were generated to supplement the missing background data for model training. In this study, a two-step environmental profiling approach was applied to define the spatial range for generating pseudo-absence points. First, K-means clustering was performed on the presence points based on Euclidean distance. Pseudo-absence points were then randomly generated within pixels that were highly dissimilar to the presence points in environmental characteristics. The locations of presence points were converted into a raster image, and pixels containing presence points were excluded to avoid overlapping presence and pseudo-absence points within the same pixel. Furthermore, an environ-mentally constrained method was used to select pseudo-absences. Environmental variable values were randomly extracted from a subset of presence pixels, and a 2-class K-means clusterer was trained using Euclidean distance. The entire study area was clustered to identify the group environmentally similar to presence points, and pseudo-absences were only generated in the dissimilar cluster. Finally, the valid area for pseudo-absence generation was determined by overlaying a water mask and the boundary of the study area. Using the K-means clustering method, grids without known presence points were classified based on their environmental similarity to the presence points, allowing the model to learn environmental differentiation features. Pseudo-absence points were generated from grids with environmental characteristics that were significantly different from the current presence points, theoretically indicating unsuitable habitats for *D. angustifolia*.

To avoid spatial autocorrelation during model training and ensure that model evaluation accurately represented the predictive capacity of *D. angustifolia*, the spatial cross-validation functionality in GEE was employed. The study area was divided into several nonoverlapping spatial blocks with a grid cell size of 5 km. Water grids within the study area were excluded to ensure that the training and validation sets were spatially independent.

#### 2.3.2 Model building

We applied a random forest classifier (Corcoran et al., 2015) and repeated spatial block cross-validation techniques (10 times) to model the habitat suitability of *D. angustifolia* (Killeen et al., 2024). A 5 × 5 km spatial block grid was defined, and the study area was randomly partitioned into 10 subsets. For each partition, 70% of the data was used for model training, whereas the remaining 30% was used for model validation to ensure spatial independence between the training and validation datasets. Spatial blocks were delineated within the defined boundaries of the Tumen River transboundary basin. In each iteration, the distribution points for *D. angustifolia* from the training set were used for model training, and the remaining points were used for validation. This process was re-peated 10 times, with a fixed random seed set to ensure reproducibility of the “grid partition-model training-validation” procedure in each iteration. The final model performance was evaluated by averaging the results from all 10 iterations to reduce errors. To balance the datasets for model fitting and validation, an equal number of pseudo-absence points was generated. The performance of the random forest classifier is believed to improve when balanced datasets (Barbet-Massin et al., 2012) (**Figure 2**, STEP 2).

For each training dataset, we implemented a random forest model comprising 500 decision trees. Subsample sets were randomly drawn with replacement from 62 distribution data points. The size of each subsample was equal to that of the original dataset, and each decision tree was trained using a unique subsample to ensure diversity among the trees. During the node-splitting process for each decision tree, three environmental variables were randomly selected. The minimum sample size for the leaf nodes was set to 1, enabling the decision trees to fit the model as closely as possible. Each tree was trained on 50% of the data from its randomly selected subsample to reduce the risk of overfitting, whereas the remaining 50% of the subsamples were retained as out-of-bag (OOB) samples for model validation.

The model produced continuous probability values ranging from 0 to 1, representing habitat suitability for *D. angustifolia*, with 0 indicating complete unsuitability and 1 indicating high suitability. Using the trained probability model, we predicted environmental variables in the study area. The suitability probability distribution was obtained by calculating the proportion of decision trees predicting “presence” for each location. This simulation process was repeated 10 times to obtain average results.

For the threshold-based classification, each decision tree was voted based on its prediction, determining the presence or absence of *D. angustifolia* in each area, with the classification threshold being set at 0.45. (A probability >0.45 indicated “presence,” while a probability <0.45 indicated “absence”). The final result was obtained by aggregating the outputs of all the decision trees.

The accuracy of each model iteration was assessed using both threshold-independent and threshold-dependent metrics. This ensured a comprehensive evaluation. We calculated the area under the receiver operating characteristic (ROC) curve (AUC). This evaluated the overall discriminative ability of the model. A higher AUC value indicates a stronger correlation between environmental factors and the *D. angustifolia* distribution model. This shows effective predictive performance. The AUC evaluation scale was as follows: low accuracy (0.5–0.7), moderate accuracy (0.7–0.8), good accuracy (0.8–0.9), and excellent accuracy (0.9–1).

**Figure 3:**
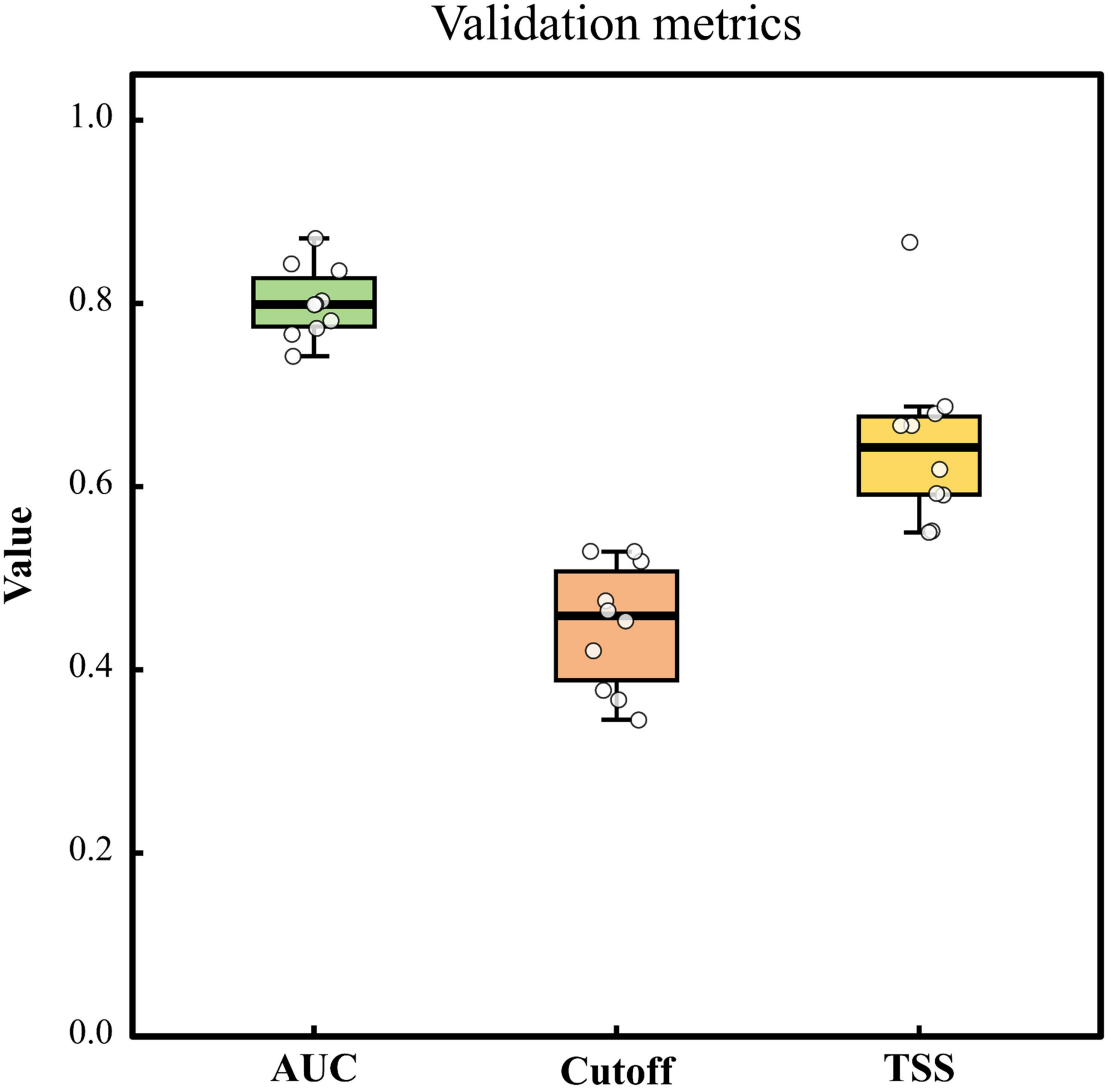
Model performance evaluation including Area under the ROC curve (AUC) True Skill Statistic (TSS) and Optimal cutoff threshold values.

Furthermore, we used the True Skill Statistic (TSS) to supplement our evaluation. This addresses the known limitations of AUC in species distribution models. TSS is a threshold-dependent metric. It considers both sensitivity and specificity. Its value ranges from -1 to +1. A TSS value greater than 0.6 indicates good predictive performance (Allouche et al., 2006). The results from 10 repeated runs of the GEE-SDM random forest model showed an average AUC of 0.801 and an average maximum TSS of 0.647. This indicates good model accuracy and high prediction reliability (**Figure 3**). Using the random forest SDM, we assessed the distribution of suitable ecological areas for *D. angustifolia* in the Tumen River transboundary basin.

Subsequently, we reclassified the final habitat suitability map into a binary potential distribution map based on the average results of the random forest model. This binary approach was used to identify the regions where potential habitats for *D. angustifolia* are present or absent. Based on the mapping relationship between the current *D. angustifolia* presence points and the environmental variables learned by the RF model, we constructed a complete SDM. We then provided environmental variable data corresponding to different emission scenarios for the 2050s and generated predictions for the future habitat distribution of *D. angustifolia*.

Based on the ensemble results from the 500 decision trees in the random forest SDM, we calculated the probability of each grid representing a potentially suitable habitat for *D. angustifolia*. The GEE output was preprocessed in ArcGIS 10.8 by removing grids located within urban construction areas. Subsequently, using the habitat suitability values for each geographic unit obtained from the random forest SDM, we classified the suitability of these units using a natural break classification method (Nan et al., 2024). Suitable habitats for *D. angustifolia* were categorized into four classes: unsuitable (0–0.45), low suitability (0.45–0.57), moderate suitability (0.57–0.71), and high suitability (0.71–1). Area calculations for each suitability class were performed using R.

Furthermore, using R and ArcGIS 10.8, we applied the control variable method to compare changes in habitat suitability for *D. angustifolia* between the present period and the 2050s under different shared socioeconomic pathway (SSPs) emission scenarios. We also analyzed changes in the binary presence/absence results for habitats under the combined impacts of climate change and land-use change (**Figure 2**, STEP 3).

## 3 Results

### 3.1 Current potential habitat distribution of *D. angustifolia*

**Figure 4:**
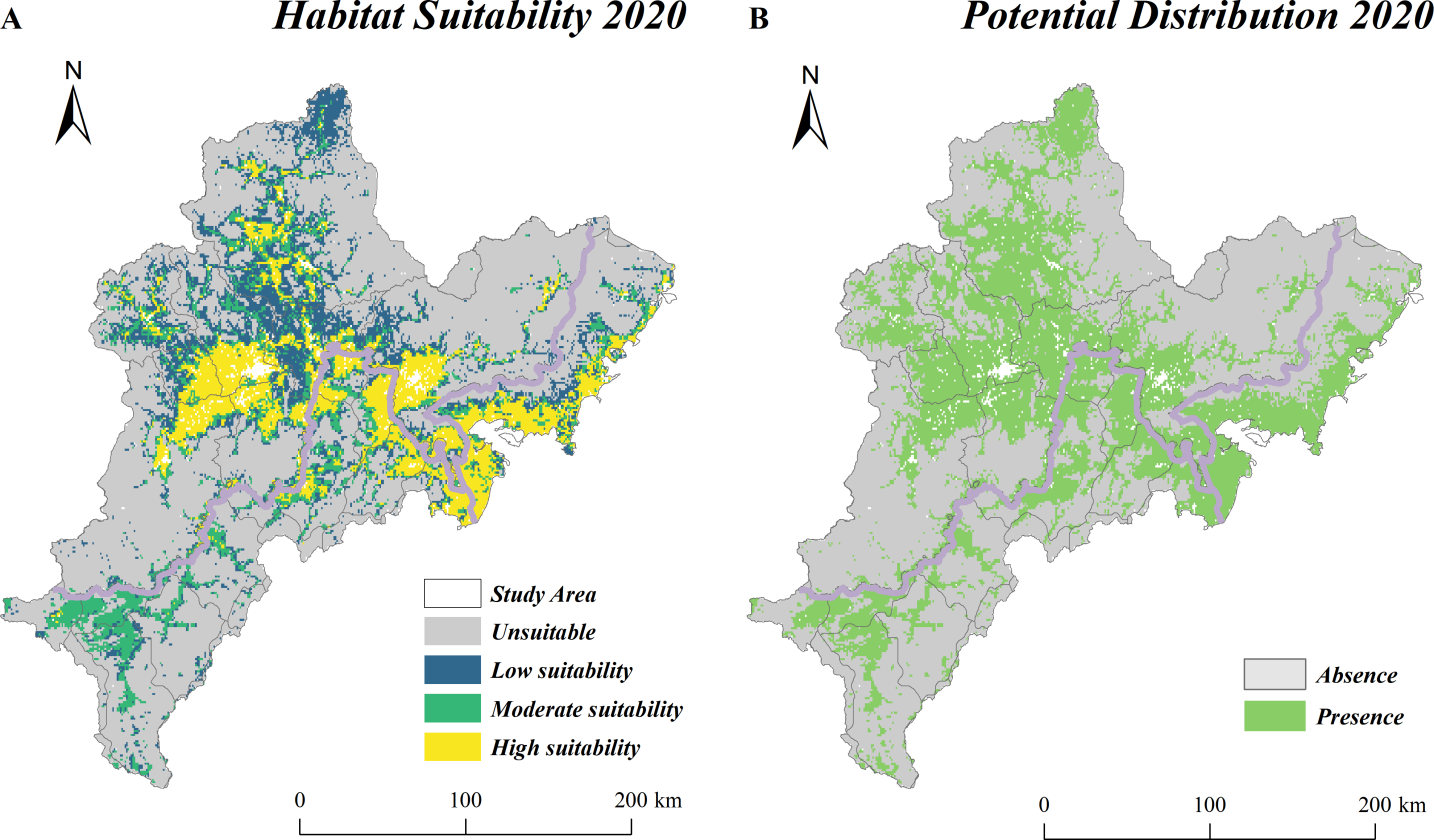
Current ecological suitability and presence–absence distribution of Deyeuxia angustifolia. **(A)** Predicted distribution map of current ecological suitability. **(B)** Binary presence–absence map under current climatic conditions.

The potential distribution predictions for *D. angustifolia* are represented as continuous output values (**Figure 4A**), with higher values indicating more favorable conditions for the survival of the species. Suitable habitats for *D. angustifolia* were predominantly located in the Russian portion of the study area. In China, suitable habitats were primarily observed in the northwest of Wangqing County, northern parts of Helong City, and northern areas of Antu County in Jilin Province. A sporadic distribution was also observed in southern Hunchun City, central Longjing City, and Tumen City. In Russia, suitable habitats are mainly concentrated in the Hassan District, whereas in DPRK, they were primarily observed in the Ryanggang and Jagang provinces.

The area and proportion of suitable habitats within the Tumen River transboundary basin were as follows: high, moderate, low, and unsuitable habitats covered 5,066.27 km² (13.72%), 4,347.49 km² (11.78%), 5,414.58 km² (14.67%), and 22,086.41 km² (59.83%), respectively. We designated all areas, except for unsuitable habitats, as presence zones for *D. angustifolia* to facilitate the comparison of presence and absence (**Figure 4B**).

### 3.2 Distribution and areal changes of potentially suitable habitats for *D. angustifolia* in 2050

**Figure 5:**
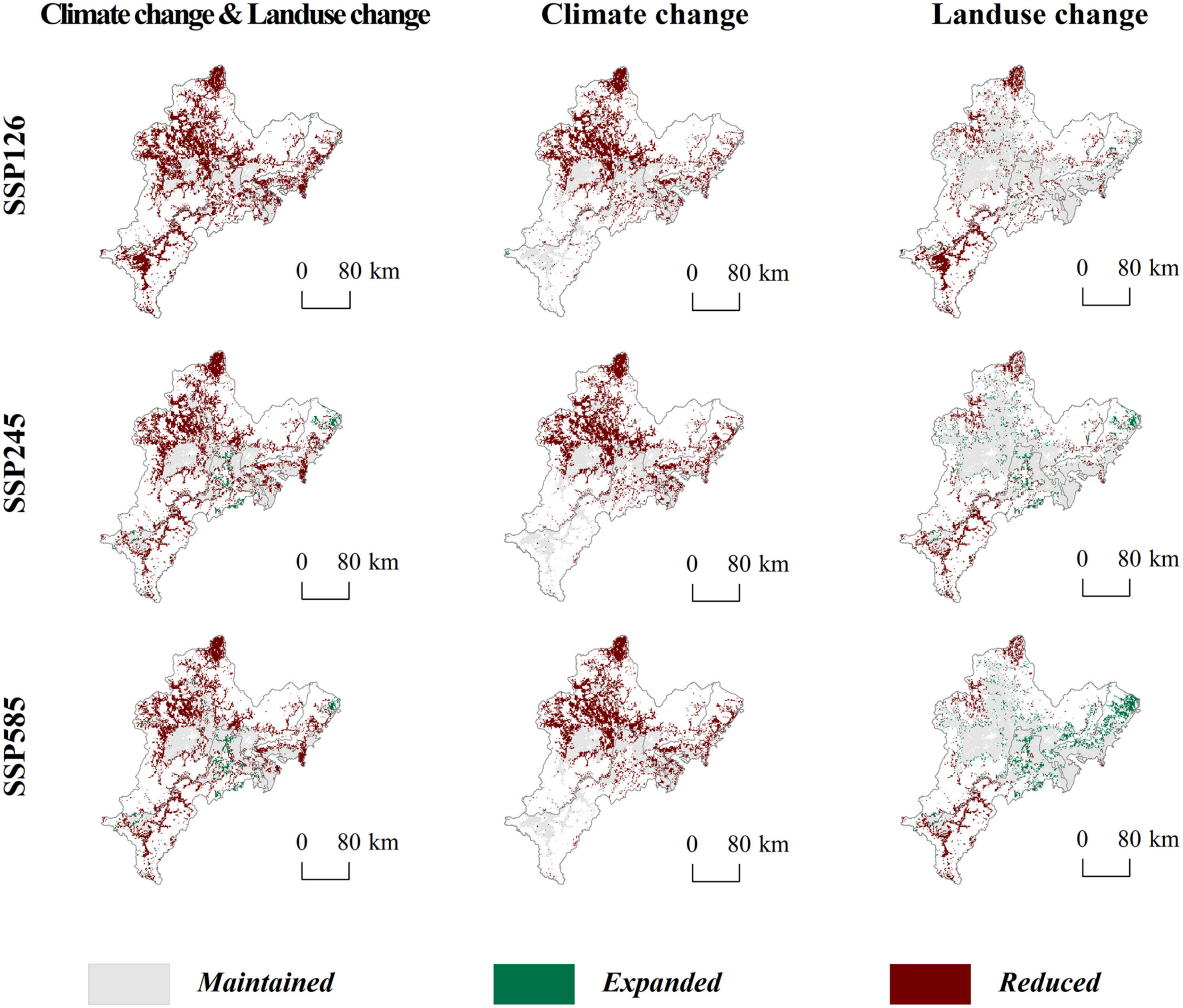
Predicted distribution changes of *D. angustifolia* under different climate- and land-use-change scenarios in 2050. Green area indicates expansion, red area indicates reduction, and light colors indicate maintained suitable areas under SSP126, SSP245, and SSP585 scenarios.

Under the combined effects of climate change and land-use change, the distribution of *D. angustifolia* habitats exhibited significant spatiotemporal variation across different future emission scenarios. As the emissions concentration increased, the areas of habitat maintenance and expansion gradually increased, whereas the area of habitat loss decreased (**Figure 5**). Under the low-emission scenario (SSP126), the area of habitat maintenance was 5,326.63 km², expansion was 94.61 km², and loss was 9,305.99 km². Under the medium emission scenario (SSP245), the area of maintenance increased to 7,330.52 km², expansion increased to 637.64 km², and loss decreased to 7,302.09 km². Under the high emission scenario (SSP585), maintenance increased to 7,753.86 km², expansion increased to 711.9 km², and loss decreased to 6,878.75 km².

Spatially, SSP126 showed the most substantial loss, which was mainly concentrated in the western and northern regions of Wangqing County, Antu County, and Tumen City on the Chinese side of the basin, as well as in the Ryanggang region of DPRK and Hassan District of Russia. Under SSP585, the largest areas of expansion and maintenance occurred, with expansion concentrated in the northern part of Hamgyongbuk-do and Huicheon-si in DPRK, and western part of Dahongtuan County and coastal regions of the Hassan District in Russia. Maintenance areas were primarily distributed in Tumen, Longjing, southern Yanji, and northern Helong cities in China, as well as in the northern part of Ryanggang and southern coastal areas of Hassan in DPRK.

In scenarios considering only climate change, the area of habitat expansion increased as the emission concentration increased, whereas the spatial distribution characteristics of the maintenance and loss areas exhibited varying degrees of differences. Under SSP126, maintenance areas were 8,167.4 km², expansion areas were 47.39 km², and loss areas were 6,465.21 km². Under SSP245, maintenance areas decreased to 7,981.38 km², expansion areas decreased to 5.11 km², and loss areas increased to 6,651.23 km². Under SSP585, maintenance areas were 7,986.43 km², expansion areas were 5.11 km², and loss areas were 6,646.18 km².

Spatially, SSP245 resulted in the most substantial loss, concentrated in the eastern part of Antu County, central Yanji City, central Tumen City, and both sides of Wangqing County, with scattered losses in the northern part of Ryanggang and coastal edges of Hassan. Under SSP126, the maintenance areas were primarily concentrated in the northern part of Helong City, central Longjing City, and southern Yanji City, as well as in the northern part of Ryanggang and coastal areas of Hassan.

In scenarios considering only land use change, the area of habitat expansion increased as the emission concentration increased; however, the spatial distribution characteristics of maintenance and loss areas still showed varying degrees of difference. Under SSP126, maintenance areas were 10,682.79 km², expansion areas were 453.02 km², and loss areas were 3,949.82 km². Under SSP245, maintenance areas increased to 11,840.31 km², expansion areas increased to 1,298.85 km², and loss areas decreased to 2,792.3 km². Under SSP585, maintenance areas were 12,290.06 km², expansion areas increased to 2,226.64 km², and loss areas were 2,342.55 km².

Spatially, under SSP126, the greatest loss was concentrated in the northern part of Hamgyongbuk-do in DPRK. Under SSP245, the largest areas of maintenance occurred in Tumen City, Hunchun City, central Wangqing County, and central Helong City, with concentrations in the northern part of Ryanggang and the coastal areas of Hassan. Under SSP585, the largest expansion occurred in the northern part of Ryanggang, with scattered expansion along the coastal area of Hassan. Overall, under land-use change scenarios, the expansion area of *D. angustifolia* was relatively high, although the total suitable habitat area showed a decreasing trend.

**Figure 6:**
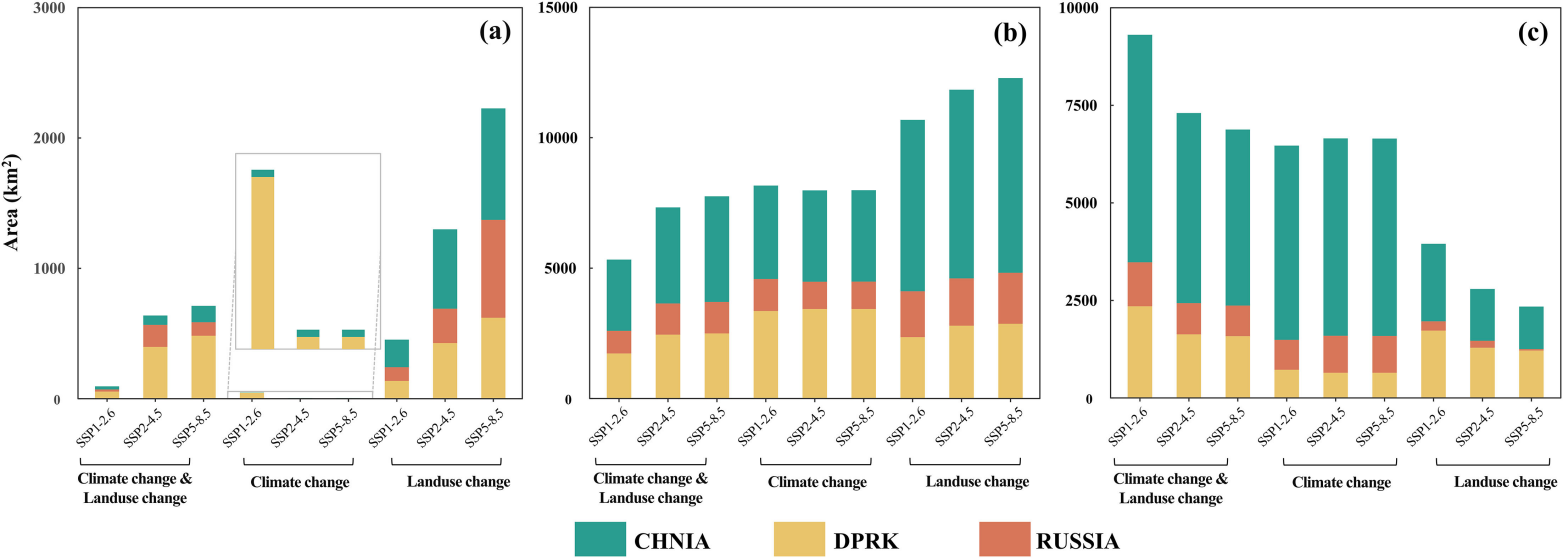
National-scale distribution changes of *D. angustifolia* under different scenarios in 2050. Bars represent areas of potential distribution expansion, maintenance, and reduction in China, DPRK, and Russia, including expansion area **(a)**, maintenance area **(b)**, and reduction area **(c)**.

The distribution of habitat maintenance, expansion, and loss for *D. angustifolia* across the basins in China, DPRK, and Russia exhibited distinct spatiotemporal variations, with a general trend of increasing habitat maintenance and expansion and decreasing habitat loss as emission concentrations increased (**Figure 6**). Changes in habitats in China were particularly pronounced. Under the combined effects of climate change and land-use change, in the low-emission scenario (SSP126), the area of habitat maintenance was 2,730.6 km², expansion was 24.08 km², and loss was 5,829.47 km². As emission concentrations increased, the area of expansion gradually increased, with SSP245 and SSP585 exhibiting increases of 47.09 and 101.85 km², respectively, compared to that of SSP126. Similarly, the area of loss gradually decreased, with reductions of 956.69 and 1,318.65 km² under SSP245 and SSP585, respectively.

In scenarios considering only climate change, under SSP126, the areas of habitat maintenance, expansion, and loss were 3,584.43, 1.9, and 4,975.63 km², respectively. As emission concentrations increased, the expansion area remained unchanged, whereas the area of loss gradually increased, with increases of 80.83 and 80.82 km² under SSP245 and SSP585, respectively.

In scenarios considering only land use change, under SSP126, the areas of habitat maintenance, expansion, and loss were 6,572.04, 211.66, and 1,988.03 km², respectively. As emission concentrations increased, the expansion area gradually increased, with increases of 396.9 and 642.89 km² under SSP245 and SSP585, respectively. The area of loss gradually decreased, with reductions of 661.56 and 897.76 km² under SSP245 and SSP585, respectively.

The trends observed in DPRK and Russia were generally consistent with those in China. However, in the climate change scenarios, the loss areas in DPRK and Russia decreased as emission concentrations increased; in the land-use change scenarios, the loss area in Russia also decreased with increasing emission concentrations. Overall, with increasing emission concentrations, the areas of habitat maintenance and expansion increased, whereas the areas of loss significantly decreased across the three countries.

**Figure 7:**
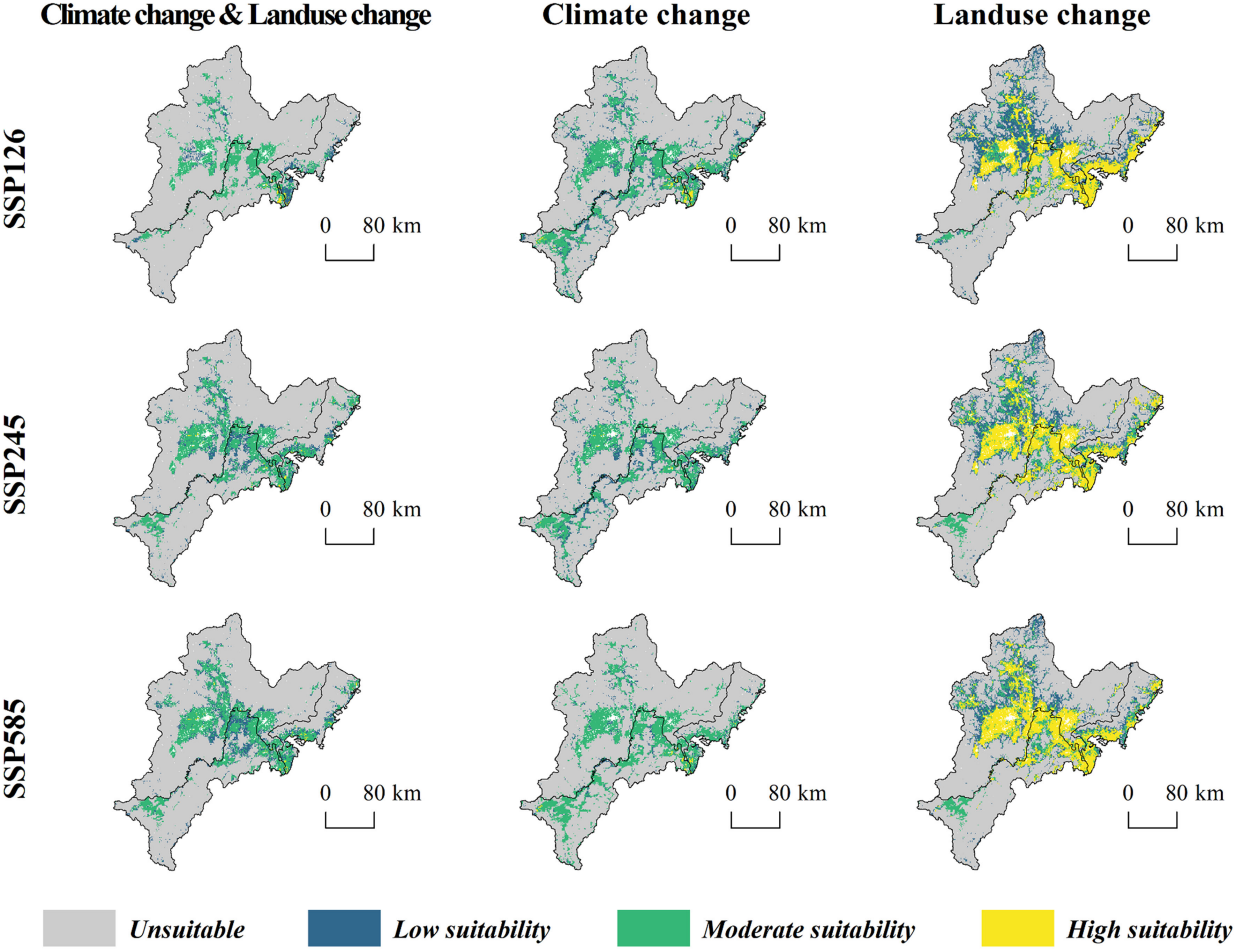
Potentially suitable habitat distribution of *D. angustifolia* in 2050 under multiple scenarios.

The changes in the suitability rankings of *D. angustifolia* habitats across different future scenarios exhibited significant spatiotemporal variations (**Figure 7**). Under the combined effects of climate and land-use change, the low-emission scenario (SSP126) had the most pronounced suppressive effect on the distribution of *D. angustifolia*. In this scenario, the area of unsuitable habitats was 31,686.36 km², accounting for 85.84% of the total basin area, whereas the total area of moderately and highly suitable habitats was only 4,140.41 km², representing a 56.02% decrease from the current state. Under SSP245 and SSP585, the areas of unsuitable habitat increased by 30.41% and 28.23%, respectively.

In the SSP585 scenario, the total area of moderately and highly suitable habitats was the largest across all three emission scenarios, reaching 6,266.34 km². However, compared to the current situation, this area decreased by 33.43%. Under climate change conditions, all three emission scenarios severely limited the distribution of *D. angustifolia*. In the SSP585 scenario, the area of unsuitable habitats increased to 28,754.26 km², representing 77.89% of the total basin area. The areas of low suitability habitats decreased as emission concentrations increased, whereas the total area of moderately and highly suitable habitats was the highest across all scenarios, at approximately 7,165.61 km². Despite this, it still experienced a significant reduction of 23.88% compared to the current state.

**Figure 8:**
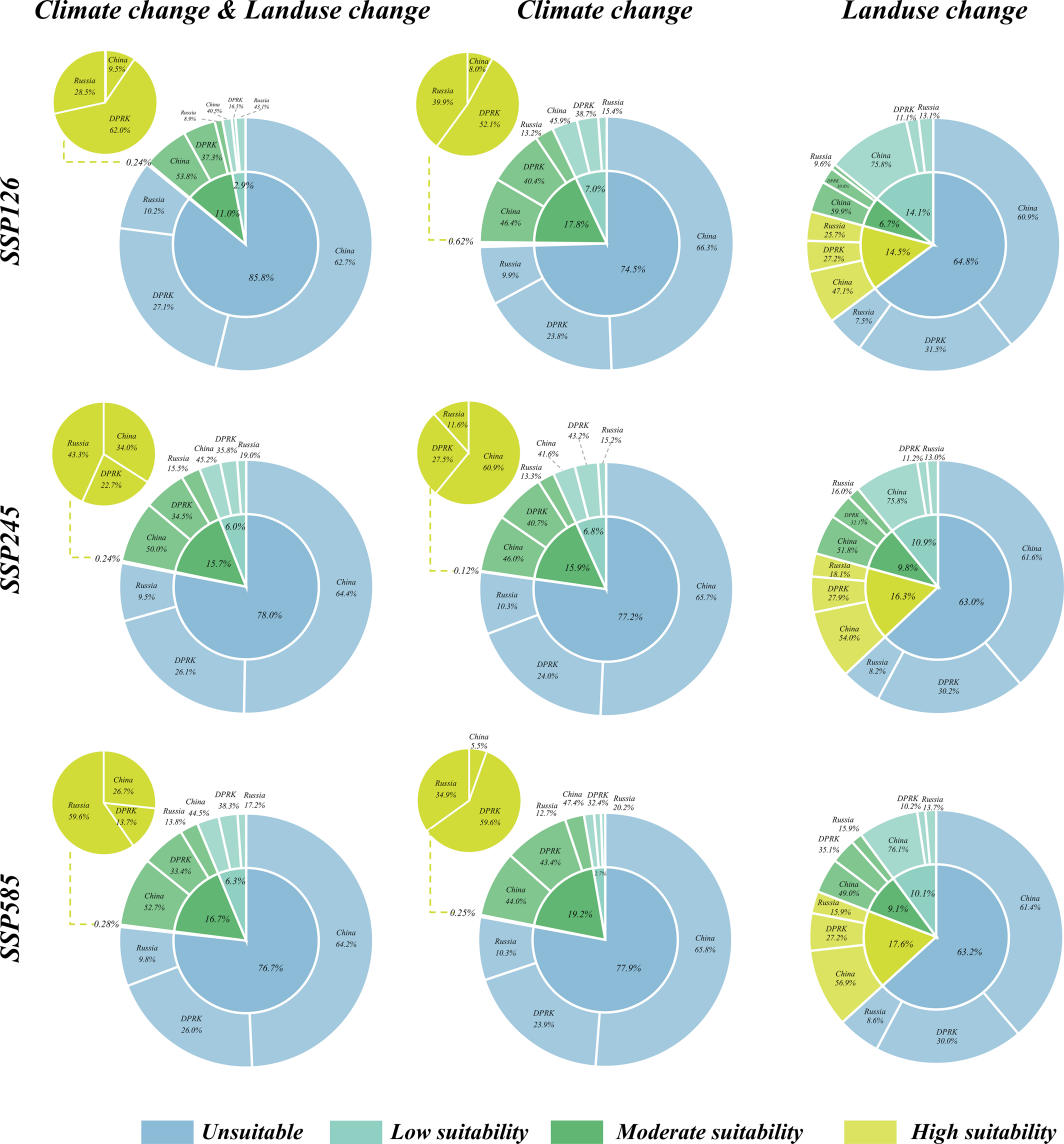
National distribution of suitable areas of *D. angustifolia* under different scenarios in 2050.

In scenarios considering only land use change, the SSP126 scenario relatively suppressed the suitable distribution range of *D. angustifolia*, with the area of unsuitable habitats reaching 23,905.2 km², an increase of 8.23% compared to that of the current state. The area of highly suitable habitats increased by 5.43%, reaching 5,341.12 km². Under the SSP245 and SSP585 scenarios, the areas of moderately suitable habitats decreased by 16.45% and 22.81%, respectively, whereas the areas of highly suitable habitats increased by 18.79% and 28.01%, respectively (**Figure 8**; **Supplementary Table 4**).

## 4 Discussion

### 4.1 Relative contributions of environmental variables

**Figure 9:**
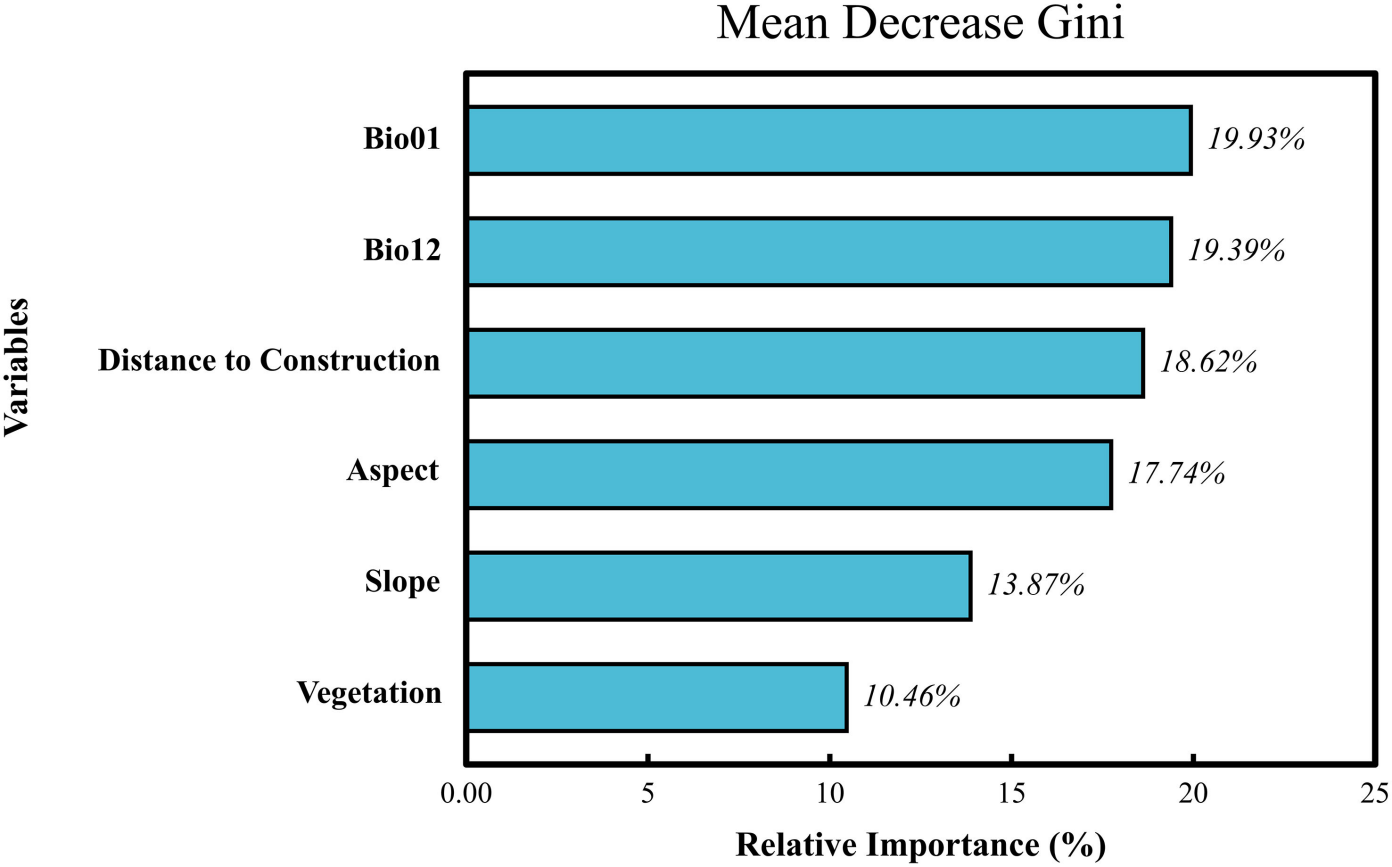
Relative proportion of environmental variable importance based on Mean Decrease Gini.

Prior to evaluating the spatial distribution patterns, we quantified the relative contribution of each environmental variable. The Mean Decrease Gini values were standardized into relative percentage contributions (**Figure 9**). Mean annual temperature (Bio01) and annual precipitation (Bio12) contributed 19.93% and 19.39%, respectively. Regional hydrothermal conditions fundamentally constrain the macroscopic distribution of this species. Temperature regulates the plant growing season, while precipitation directly controls hydrological fluctuations in wetlands (Erwin, 2009). Distance to built-up areas was the third most important driving factor (18.62%), which highlights the profound impact of human activities on wetland ecosystems. Urban construction activities typically alter local drainage and induce nutrient enrichment (Jiang et al., 2020), which promotes the expansion of highly competitive species such as *D. angustifolia* into degraded wetlands. Topographic factors also played a critical role, with aspect and slope contributing 17.74% and 13.87%, respectively. Microtopography determines local solar radiation and soil water retention, creating essential microhabitats for the survival of wetland plants (Moeslund et al., 2013). Vegetation type had a relatively low contribution (10.46%). These underlying driving mechanisms logically explain the subsequent spatial distribution changes under different scenarios.

### 4.2 Effects of climate change and land use on *D. angustifolia*

Under current conditions, *D. angustifolia* was primarily distributed in the central and northern regions of the Tumen River Basin on the Chinese side, the southern region on the Korean side, and along the coastal areas on the Russian side. Its distribution was commonly associated with rivers, forested wetlands, and paddy fields, which is consistent with the findings of Zong et al. (2022). This suggests that the species tends to occupy moist landforms with sufficient water availability, matching its preference for wet ecological niches. On the Chinese side of the basin, suitable habitats for *D. angustifolia* were concentrated in the central and northern regions, whereas unsuitable areas were predominantly found in the west and south. This distribution pattern was primarily due to the overall topography of the Chinese side of the basin, which slopes from west to east. The southwestern, northwestern, and northeastern areas are more prone to soil erosion, leading to accelerated moisture loss from the wetlands, which may hinder the continued growth of *D. angustifolia*. By contrast, the coastal regions of Russia, such as the Vladivostok area, present favorable water conditions that provide a more stable habitat for the species. Our predictions indicated that 1,086.08 km² (26.52%) of the total suitable area in the Russian part of the basin is a highly suitable habitat for *D. angustifolia*. Additionally, this area is subject to relatively minimal human disturbance and provides favorable natural conditions. Its unsuitable area was the smallest among the Chinese, Korean, and Russian sections of the basin, covering only 2,002.44 km². Furthermore, *D. angustifolia* demonstrates strong growth potential in agricultural fields, particularly paddy fields, which suggests interspecific competition with rice. Between 1976 and 2016, the wetland area of the Tumen River Basin generally decreased, with significant reductions in river and marsh wetlands and the exacerbation of paddy field degradation (Zhang et al., 2020). This finding is consistent with the results of the present study, in which the most suitable habitats for the species were concentrated in areas of wetland degradation.

In future scenarios, climate and land use changes are expected to notably affect the potential distribution of *D. angustifolia*, with an overall trend of substantial suppression. Under climate change scenarios, the distribution area of *D. angustifolia* was projected to decrease from 24.59% to 30.19%. This notable reduction is primarily driven by disruptions in the spatial and temporal distribution of precipitation and an increase in extreme precipitation events, both of which can destabilize the moisture supply in wetlands, thereby stressing species growth. Previous studies have shown that climate warming can extend the growing season of plants by several weeks, thereby increasing their photosynthetic carbon uptake and aboveground biomass. Therefore, under high-emission scenarios, the habitat of *D. angustifolia* may expand (Richardson et al., 2018). This finding is highly consistent with the conclusions of our study. Notably, under higher emission scenarios (SSP585), although the overall suitable area still decreased, the area of moderately and highly suitable habitats increased by 5.34% and 21.39% compared to those of SSP126 and SSP245, respectively, indicating a weakening of the suppression effect. This may be because of increased atmospheric CO_2_ concentrations, which enhance photosynthesis in plants, increase physiological activity, and partially mitigate the negative effects of climate change. This finding is consistent with the conclusions of ecological risk studies on wetland vegetation on the Sanjiang Plain (Fu et al., 2020).

In land-use change scenarios, the reduction in *D. angustifolia* distribution was primarily concentrated in the upstream Tumen River region and around agricultural lands on the Korean side, with suitable habitat areas declining by 7.85%–12.27%. The reduction in *D. angustifolia* distribution in the upper reaches of the Tumen River is associated with changes in land use, which affected the habitat of the species. In contrast, excessive agricultural expansion results in severe soil organic carbon loss. However, excessive deforestation substantially reduces the capacity to intercept pollutants emitted by human activities, leading to the accumulation of air and water pollutants in species’ habitats (Lim, 2021). Notably, under land-use change scenarios with higher emission concentrations (SSP245 and SSP585), the areas of highly suitable habitats increased (by 18.79% and 28.01%, respectively). With the advancement of urbanization and policies, such as returning farmland to forests, forested areas are increasing, while cultivated land is decreasing. Additionally, the trend of reverse urbanization in Northeast China, leading to population loss, has converted certain farmlands into abandoned fields. These changes indicate that the growth of *D. angustifolia* may be promoted under strong human intervention.

Under the combined effects of climate change and land use change, the distribution area of *D. angustifolia* is expected to decline by 42.05–64.74%, with the most significant decline occurring under the SSP126 scenario. As the emission concentration increased, the suppression effect was mitigated, and the area of highly suitable habitats increased by 2.92% and 17.52% under the SSP245 and SSP585 scenarios, respectively, compared to that of SSP126. Furthermore, the distribution of *D. angustifolia* around agricultural lands on the Chinese side of the basin is projected to increase. Global warming, characterized by increasing temperatures and reduced precipitation, leads to a decrease in the average water level of wetlands, resulting in the loss of wetland vegetation habitats (Xiong et al., 2023). Appropriate human activities have the potential to mitigate wetland degradation to some extent. Under the SSP585 high-emission scenario accompanied by intensive land-use change, the distribution range of *D. angustifolia* is projected to expand. *D. angustifolia* is characterized by rapid reproduction, strong competitive ability, and moderate tolerance to adverse conditions such as environmental pollution. Furthermore, under high-emission pathways, water pollution induced by agricultural intensification and urban expansion, including water acidification, nitrogen deposition, and other related disturbances, becomes increasingly severe. Such pollution can significantly inhibit the growth of competing species such as *R. chrysanthum* and reduce wetland biodiversity, thereby enhancing the ecological dominance of *D. angustifolia* (Li N et al., 2023).

The expansion of *D. angustifolia* under the SSP585 scenario is fundamentally driven by its functional traits and physiological adaptability. Variable importance analysis revealed that temperature (Bio01) and precipitation (Bio12) are the primary drivers. Under the SSP585 high-emission scenario, climate warming combined with elevated CO_2_ concentrations significantly enhances the net primary productivity (NPP) and vegetation growth of this species (Wang Y. et al., 2022; Yan, 2022). Furthermore, facing future extreme precipitation fluctuations, this species exhibits remarkable phenotypic plasticity. *D. angustifolia* actively adjusts its rhizome bud number and biomass across different moisture gradients. It adopts a “guerrilla-type” clonal configuration to rapidly expand and adapt to intense hydrological changes (Li H et al., 2023).

Anthropogenic disturbance, represented by the distance to construction, is the third key driver. In scenarios involving human interference and farmland abandonment, native seed-dispersed plants usually recover slowly. However, *D. angustifolia* is a highly competitive, rhizome-based species. It uses an aggressive clonal reproduction strategy to continuously expand its underground rhizome network. This allows the plant to rapidly occupy vacant ecological niches in abandoned farmlands and degraded wetland margins (Zou et al., 2014). Therefore, the suitable habitat for *D. angustifolia* presents an expansion pattern under the high-emission (SSP585) scenario.

### 4.3 Model application

Species distribution modeling (SDM) based on the Google Earth Engine (GEE) has become increasingly sophisticated. Crego et al. developed a random forest SDM on the GEE platform to predict the potential habitat of the reticulated giraffe (Giraffa reticulata) in Kenya on a large scale, providing a foundation for habitat suitability modeling of endangered terrestrial species (Crego et al., 2024). Similarly, Rachel et al. used a GEE to model the distribution and abundance of red-billed quelea (Quelea quelea), providing scientific support for the development of adaptive management strategies for these species (Dobson et al., 2023).

In the present study, we constructed a distribution model for *D. angustifolia* based on the GEE platform. This model predicts potential distribution under climate change scenarios as well as incorporates land use change (LUC) into the analytical framework, addressing the previously limited consideration of land use and land cover change (LUCC) in previous studies (Zong et al., 2022). Because of the accelerating urbanization process, expansion of built-up areas, and destruction of wetland habitat connectivity owing to road construction, the inclusion of land-use change factors is essential. Moreover, the construction of SDMs is typically computationally intensive and complex; however, the cloud computing capabilities of GEE notably enhance model efficiency, providing a robust and scalable methodological foundation for interpreting changes in wetland species habitats.

### 4.4 Limitations

Although we developed a future distribution prediction model for *D. angustifolia* in the Tumen River Basin based on GEE, certain limitations remain. First, the species sample data may have been biased. Owing to the challenges associated with cross-border field surveys, we relied on data from the Global Biodiversity Information Facility (GBIF) and limited field sampling records. Although biased data constitute <20% of the total data and have been rigorously screened for accurate spatial coordinates, they may still affect the predictive accuracy of the model in localized areas.

To reduce multicollinearity (Pearson < 0.8), we retained highly generalized macro-variables (Bio1 and Bio12). This approach may obscure key ecophysiological drivers. The expansion of wetland species like *D. angustifolia* is usually constrained by extreme climates. Despite their functional ecological significance, we excluded these extreme variables. Previous studies show that using complex extreme bioclimatic variables does not necessarily improve model performance. Instead, it significantly increases the uncertainty of future spatial predictions (Beaumont et al., 2008; Steen et al., 2017). Under future climate change, selecting from highly correlated climate variables also amplifies prediction errors (Braunisch et al., 2013). To ensure the robustness of our future scenario simulations, we prioritized the more reliable mean variables. Future studies should aim to integrate high-confidence projections of extreme climates. This will better capture the functional ecological limits of the species.

While the Random Forest (RF) model demonstrated high accuracy and excellent computational efficiency within the GEE platform, different modeling algorithms (such as MaxEnt or Generalized Linear Models) rely on distinct statistical assumptions. This can lead to variations in the projected suitable habitats. Relying on a single model may introduce algorithm-specific uncertainties. Future research should consider employing ensemble modeling approaches. By integrating multiple species distribution models, we can further reduce model-specific biases and improve the overall robustness of habitat predictions.

There are still limitations in generating pseudo-absence points in this study. K-means clustering was used to place pseudo-absences in areas with distinct environmental features (Crego et al., 2022). This method helps the model quickly distinguish suitable habitats. However, this method may place pseudo-absences into environmentally distant areas. This partly simplifies the classification task for the random forest model. Therefore, it may artificially inflate evaluation metrics like AUC. This can lead to overly optimistic accuracy estimates. This methodological flaw affects the credibility of the spatial simulation results. This is especially evident at the margins of the species range or invasion fronts. Future studies should compare multiple pseudo-absence generation strategies.

Furthermore, future research could improve their spatial resolution, and incorporate more detailed land-use change data to enhance model performance, thereby providing more scientifically robust and operational support for the conservation and management of the Tumen River wetland.

## Data Availability

The original contributions presented in the study are included in the article/**Supplementary Material**. Further inquiries can be directed to the corresponding author.
