# Supplementary material for: Predicting the distribution of Deyeuxia angustifolia habitats in the Tumen River Basin due to climate and land-use changes
Source: Front Plant Sci. 2026 Jun 26;17:1865223. doi: 10.3389/fpls.2026.1865223 (PMC13350318; doi:10.3389/fpls.2026.1865223)
Supplement: Supplementary file 1 [file DataSheet1.pdf]

frontiers

Supplementary Material

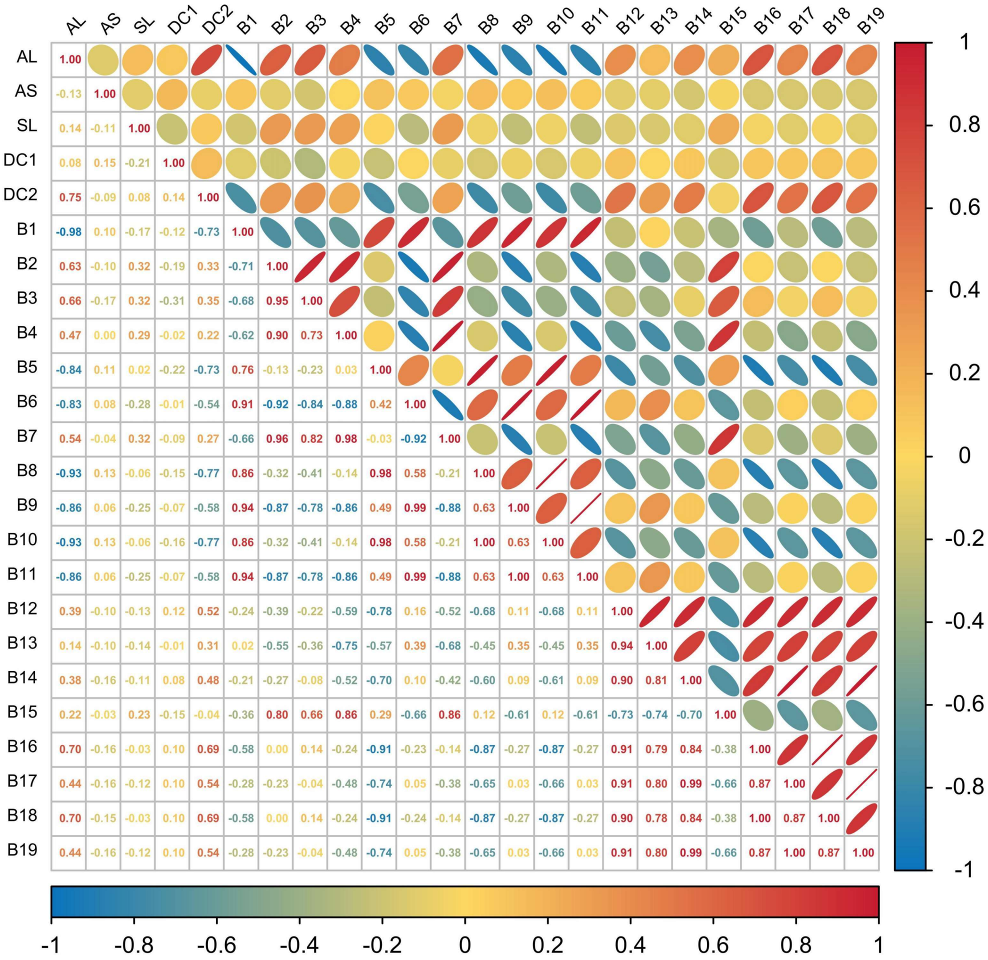

Figure S1. Results of correlation analysis of 24 environmental factors.

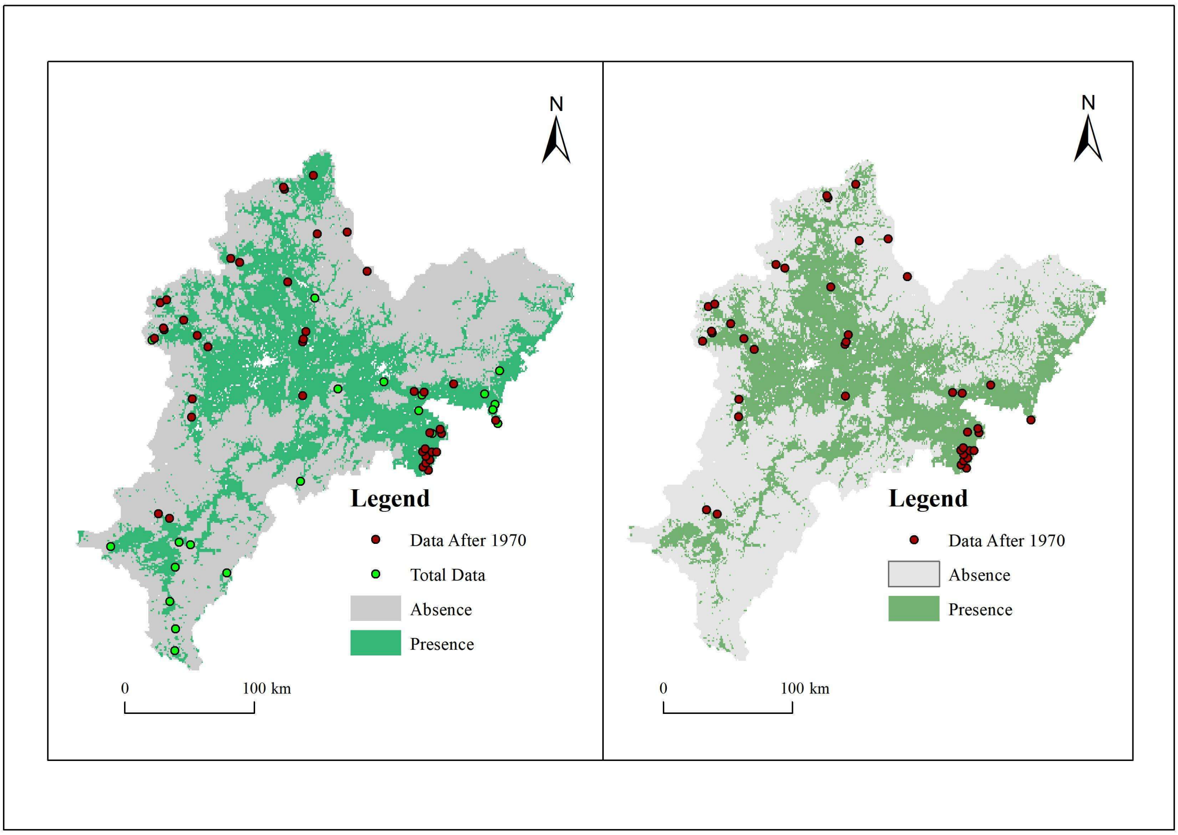

Figure S2. Sensitivity comparison of current *Deyeuxia angustifolia* distribution patterns with and without historical occurrence data

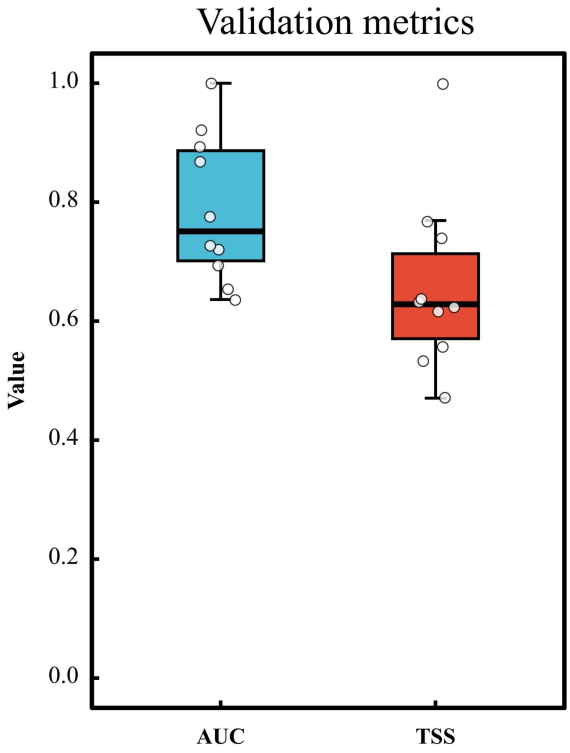

Figure S3.Model evaluation metrics for the sensitivity test

Table S1. Environment category.

Category	Variable name	Code	Description	Raw data
Terrain factors	Altitude	Al	Elevation of site area(m)	Terrain data
	Aspect	As	0 flat (-1-0),1 North (315-360,0-45),2 East (45-135),3 South (135-225),4 West (225-315)	
	Slope	Sl	Slope of site area(°C)	
Climatic factors	Bio1	B1	Annual mean temperature(°C)	Climate data
	Bio2	B2	Mean diurnal range (Mean of monthly (max temp - min temp))(°C)	
	Bio3	B3	Isothermality (Bio02/Bio07*100)	
	Bio4	B4	Temperature seasonality (Standard deviation * 100) (°C)	
	Bio5	B5	Max temperature of warmest month (°C)	
	Bio6	B6	Min temperature of coldest month (°C)	
	Bio7	B7	Temperature annual range (Bio05-Bio06) (°C)	
	Bio8	B8	Mean temperature of wettest quarter (°C)	
	Bio9	B9	Mean temperature of driest quarter (°C)	
	Bio10	B10	Mean temperature of warmest quarter (°C)	
	Bio11	B11	Mean temperature of coldest quarter (°C)	
	Bio12	B12	Annual precipitation (mm)	
	Bio13	B13	Precipitation of wettest month (mm)	
	Bio14	B14	Precipitation of driest month (mm)	
	Bio15	B15	Precipitation seasonality (Coefficient of Variation)	
	Bio16	B16	Precipitation of wettest quarter (mm)	
	Bio17	B17	Precipitation of driest quarter (mm)	
	Bio18	B18	Precipitation of warmest quarter (mm)	
	Bio19	B19	Precipitation of coldest quarter (mm)	
Natural factors	Vegetation	-	1(Evergreen Needleleaf Forests),2(Deciduous Needleleaf Forests),3(Deciduous Broadleaf Forests),4(Mixed Forests), 5(Woody Savannas),6(Savannas),7(Grasslands),8(no vegetation)	Landuse data
Social factors	Distance to construction	Dc1	Distance to construction (m)	Landuse data
	Distance to river	Dc2	Distance to river (m)	

Table S2. Details of the global climate models use in this study.

GCM	SSP1-2.6	SSP2-4.5	SSP5-8.5
ACCESS-CM2	T	T	T
BCC-CSM2-MR	F	F	F
CMCC-ESM2	T	T	T
EC-Earth3-Veg	T	T	T
FIO-ESM-2-0	T	T	F
GFDL-ESM4	T	N/A	N/A
GISS-E2-1-G	T	T	T
HadGEM3-GC31-LL	T	T	T
INM-CM5-0	T	T	T
IPSL-CM6A-LR	T	T	T
MIROC6	T	T	T
MPI-ESM1-2-HR	T	T	T
MRI-ESM2-0	T	T	T
UKESM1-0-LL	F	T	F

Table S3. Occurrence points

Name of the project	Latitude	Longitude	Original source	Time range
Deyeuxia angustifolia	42.08	128.72	Acquired from fieldwork	After 1970
Deyeuxia angustifolia	42.05	128.80	Acquired from fieldwork	After 1970
Deyeuxia angustifolia	42.41	130.72	Acquired from fieldwork	After 1970
Deyeuxia angustifolia	42.41	130.76	Acquired from fieldwork	After 1970
Deyeuxia angustifolia	43.02	128.69	Acquired from fieldwork	After 1970
Deyeuxia angustifolia	43.01	128.67	Acquired from fieldwork	After 1970
Deyeuxia angustifolia	42.70	128.96	Acquired from fieldwork	After 1970
Deyeuxia angustifolia	42.98	129.08	Acquired from fieldwork	After 1970
Deyeuxia angustifolia	43.02	129.78	Acquired from fieldwork	After 1970
Deyeuxia angustifolia	43.00	129.77	Acquired from fieldwork	After 1970
Deyeuxia angustifolia	43.07	128.76	Acquired from fieldwork	After 1970
Deyeuxia angustifolia	43.08	128.75	Acquired from fieldwork	After 1970
Deyeuxia angustifolia	43.23	128.78	Acquired from fieldwork	After 1970
Deyeuxia angustifolia	43.21	128.73	Acquired from fieldwork	After 1970
Deyeuxia angustifolia	43.43	129.31	Acquired from fieldwork	After 1970
Deyeuxia angustifolia	43.45	129.25	Acquired from fieldwork	After 1970
Deyeuxia angustifolia	43.04	129.00	Acquired from fieldwork	After 1970
Deyeuxia angustifolia	43.06	129.80	Acquired from fieldwork	After 1970
Deyeuxia angustifolia	43.32	129.66	Acquired from fieldwork	After 1970
Deyeuxia angustifolia	43.38	130.25	Acquired from fieldwork	After 1970
Deyeuxia angustifolia	43.59	130.10	Acquired from fieldwork	After 1970
Deyeuxia angustifolia	43.82	129.64	Acquired from fieldwork	After 1970
Deyeuxia angustifolia	43.83	129.63	Acquired from fieldwork	After 1970
Deyeuxia angustifolia	43.89	129.85	Acquired from fieldwork	After 1970
Deyeuxia angustifolia	41.78	128.84	Acquired from GBIF and literature	Before 1970
Deyeuxia angustifolia	41.60	128.80	Acquired from GBIF and literature	Before 1970
Deyeuxia angustifolia	41.45	128.84	Acquired from GBIF and literature	Before 1970
Deyeuxia angustifolia	41.33	128.84	Acquired from GBIF and literature	Before 1970
Deyeuxia angustifolia	41.75	129.22	Acquired from GBIF and literature	Before 1970
Deyeuxia angustifolia	41.90	128.36	Acquired from GBIF and literature	Before 1970
Deyeuxia angustifolia	41.92	128.87	Acquired from GBIF and literature	Before 1970
Deyeuxia angustifolia	41.91	128.95	Acquired from GBIF and literature	Before 1970
Deyeuxia angustifolia	42.25	129.76	Acquired from GBIF and literature	Before 1970
Deyeuxia angustifolia	42.31	130.70	Acquired from GBIF and literature	After 1970

Deyeuxia angustifolia	42.33	130.66	Acquired from GBIF and literature	After 1970
Deyeuxia angustifolia	42.41	130.65	Acquired from GBIF and literature	After 1970
Deyeuxia angustifolia	42.37	130.71	Acquired from GBIF and literature	After 1970
Deyeuxia angustifolia	42.39	130.68	Acquired from GBIF and literature	After 1970
Deyeuxia angustifolia	42.43	130.67	Acquired from GBIF and literature	After 1970
Deyeuxia angustifolia	42.35	130.68	Acquired from GBIF and literature	After 1970
Deyeuxia angustifolia	42.60	128.96	Acquired from GBIF and literature	After 1970
Deyeuxia angustifolia	42.75	130.03	Acquired from GBIF and literature	Before 1970
Deyeuxia angustifolia	42.72	129.77	Acquired from GBIF and literature	Before 1970
Deyeuxia angustifolia	42.63	130.63	Acquired from GBIF and literature	Before 1970
Deyeuxia angustifolia	42.74	130.59	Acquired from GBIF and literature	After 1970
Deyeuxia angustifolia	42.79	130.37	Acquired from GBIF and literature	Before 1970
Deyeuxia angustifolia	42.72	130.65	Acquired from GBIF and literature	Before 1970
Deyeuxia angustifolia	42.51	130.79	Acquired from GBIF and literature	After 1970
Deyeuxia angustifolia	42.67	131.18	Acquired from GBIF and literature	Before 1970
Deyeuxia angustifolia	42.64	131.17	Acquired from GBIF and literature	Before 1970
Deyeuxia angustifolia	42.73	131.11	Acquired from GBIF and literature	Before 1970
Deyeuxia angustifolia	42.51	130.71	Acquired from GBIF and literature	After 1970
Deyeuxia angustifolia	42.78	130.88	Acquired from GBIF and literature	After 1970
Deyeuxia angustifolia	42.57	131.21	Acquired from GBIF and literature	Before 1970
Deyeuxia angustifolia	42.73	130.67	Acquired from GBIF and literature	After 1970
Deyeuxia angustifolia	42.58	131.19	Acquired from GBIF and literature	After 1970
Deyeuxia angustifolia	42.53	130.78	Acquired from GBIF and literature	After 1970
Deyeuxia angustifolia	42.51	130.73	Acquired from GBIF and literature	Before 1970
Deyeuxia angustifolia	42.85	131.22	Acquired from GBIF and literature	Before 1970
Deyeuxia angustifolia	43.12	128.90	Acquired from GBIF and literature	After 1970
Deyeuxia angustifolia	43.24	129.86	Acquired from GBIF and literature	Before 1970
Deyeuxia angustifolia	43.58	129.88	Acquired from GBIF and literature	After 1970

Table S4. Suitability zoning results of *Deyeuxia angustifolia* under current and future diverse scenarios

**Different scenarios and status**			**Absolute area of different scenarios and status/km2**			
			Unsuitable area	Low suitable area	Moderate suitable area	High suitable area
Current		China	13879.6836	3923.8272	1994.2111	2696.5229
		DPRK	6204.2825	931.5606	1904.918	1283.6665
		Russia	2002.4437	559.1897	448.365	1086.0819
		**TOTAL**	**22086.4099**	**5414.5775**	**4347.4941**	**5066.2713**
Climate change Landuse change	SSP1-2.6	China	19864.8499	440.1323	2181.0298	8.2327
		DPRK	8577.8307	179.2193	1513.5486	53.8291
		Russia	3243.6802	468.6301	359.072	24.6981
		**TOTAL**	**31686.3608**	**1087.9818**	**4053.6504**	**86.7599**
	SSP2-4.5	China	18560.2851	1000.5886	2902.9735	30.3976
		DPRK	7510.114	792.2382	2001.8105	20.2651
		Russia	2733.2534	421.1338	903.0629	38.6303
		**TOTAL**	**28803.6524**	**2213.9606**	**5807.8468**	**89.293**
	SSP5-8.5	China	18180.3147	1036.0525	3250.6463	27.2312
		DPRK	7359.3924	889.7639	2061.3392	13.9322
		Russia	2782.6495	400.2354	852.4002	60.7953
		**TOTAL**	**28322.3567**	**2326.0518**	**6164.3857**	**101.9587**
Climate change	SSP1-2.6	China	18237.3103	1191.207	3047.3622	18.3652
		DPRK	6544.356	1005.0216	2655.3595	119.6907
		Russia	2736.4198	398.3356	869.4988	91.8262
		**TOTAL**	**27518.0861**	**2594.5642**	**6572.2205**	**229.8821**
	SSP2-4.5	China	18723.6723	1049.9847	2693.9898	26.5979
		DPRK	6840.0996	1089.2483	2383.0474	12.0324
		Russia	2926.405	382.5035	782.1056	5.0663
		**TOTAL**	**28490.1769**	**2521.7366**	**5859.1428**	**43.6966**
	SSP5-8.5	China	18906.6914	471.1632	3111.3239	5.0663
		DPRK	6880.6298	322.3415	3066.3607	55.0957
		Russia	2966.9352	201.3843	895.4635	32.2975
		**TOTAL**	**28754.2563**	**994.889**	**7073.1481**	**92.4595**
Landuse change	SSP1-2.6	China	14561.0971	3947.8919	1471.1185	2514.1372
		DPRK	7540.5116	580.0881	751.0747	1452.7533
		Russia	1803.5926	682.6801	235.5816	1374.2261
		**TOTAL**	**23905.2013**	**5210.6601**	**2457.7749**	**5341.1166**
	SSP2-4.5	China	14316.0162	3043.5625	1882.1198	3252.5462
		DPRK	7030.0848	448.9983	1167.7755	1677.5691
		Russia	1904.9180	520.5594	582.6212	1087.9818
		**TOTAL**	**23251.019**	**4013.1202**	**3632.5166**	**6018.0971**
	SSP5-8.5	China	14328.6819	2833.9455	1642.7385	3688.8788
		DPRK	7002.2203	379.9704	1178.5414	1763.6957
		Russia	2016.376	512.3267	534.4916	1032.8861
		**TOTAL**	**23347.2782**	**3726.2426**	**3355.7715**	**6485.4606**
